# Effects of Puberty and First Insemination Parameters on Lifetime Reproductive Performance in Replacement Gilts

**DOI:** 10.3390/ani16101513

**Published:** 2026-05-15

**Authors:** Junwen Wang, Jiajian Tan, Haiqing Sun, Hongkui Wei, Jian Peng, Yuanfei Zhou

**Affiliations:** 1 College of Animal Science and Technology, Huazhong Agricultural University, Wuhan 430070, China; sxczwjw@163.com (J.W.); weihongkui@mail.hzau.edu.cn (H.W.);; 2 Center for Frontier Science in Animal Breeding and Sustainable Production, Ministry of Education, Wuhan 430070, China; 3 Guangxi Yangxiang Joint Stock Company, Guigang 537000, China; tjjggyx@sina.com (J.T.); sunhaiqing1985110@163.com (H.S.)

**Keywords:** replacement gilts, puberty parameters, first insemination parameters, lifetime reproductive performance, retention rate

## Abstract

This study investigated the influence of age, body weight, and backfat thickness at puberty and first insemination on the reproductive performance and retention rate across multiple parities in replacement gilts. A total of 3025 DanBred gilts were classified according to these factors. Their reproductive performance and survival were monitored up to the fifth parity. The estrus detection rate was 88.36%, and the success rate of insemination was 87.63%. Retention rates from the first to the fifth parity were 85.10%, 73.82%, 59.37%, 47.42%, and 36.36%, respectively. Multiple regression analysis further revealed that age at puberty was negatively associated with total born, born alive, healthy piglets, and weaned piglets across five parities (*p* < 0.01), while backfat thickness at puberty and first insemination showed consistent positive associations with these reproductive outcomes (*p* < 0.05). Gilts that reached puberty between 190 and 199 days of age had the highest total number of piglets born and weaned across five parities, whereas a delay in puberty onset significantly decreased the fifth-parity retention rate. Gilts with a backfat thickness > 15 mm at puberty exhibited the best reproductive performance, with a retention rate of 40.18%. For first insemination, gilts mated at 210–219 days (second estrus) produced more piglets, including more healthy piglets, across five parities, but delayed insemination age reduced the fifth-parity retention rate. Gilts mated at a body weight ≥ 140 kg showed better litter size performance, and those with a backfat thickness > 16 mm at first insemination had the greatest total number of piglets born and weaned. These results emphasize that optimal puberty and insemination parameters enhance long-term reproductive performance and retention in replacement gilts.

## Simple Summary

This study investigated the effects of age, body weight, and backfat thickness at puberty and first insemination on the lifetime reproductive performance and retention rate of replacement gilts. A total of 3025 DanBred gilts were monitored up to the fifth parity. The estrus detection rate and successful insemination rate were 88.36% and 87.63%, respectively. The retention rate gradually decreased from 85.10% in the first parity to 36.36% in the fifth parity. Gilts that reached puberty at 190–199 days of age and with backfat thickness > 15 mm at puberty had the highest total number of piglets born and weaned across five parities, as well as a higher retention rate. For first insemination, gilts bred at 210–219 days of age, body weight ≥ 140 kg, and backfat thickness > 16 mm produced more piglets and showed better lifetime reproductive performance. Multicollinearity diagnosis and multiple linear regression were applied to identify key predictive variables, and linear relationships with optimal thresholds were confirmed. These results demonstrate that optimizing age, body weight, and backfat thickness at puberty and first insemination can significantly improve long-term reproductive performance and retention rate in replacement gilts, providing a scientific reference for standardized rearing and management of Danbred replacement gilts in commercial pig farms.

## 1. Introduction

Reproductive efficiency in sows is a critical determinant of the economic sustainability of pig production systems. It is commonly evaluated by two key indicators: annual productivity, defined as the number of piglets weaned per sow per year (PSY), and lifetime productivity, referring to the total number of piglets weaned per sow over her entire productive life (PSL) [1]. To improve herd PSY, some producers tend to cull low-parity sows with low annual numbers of piglets born alive. However, this practice prevents sows from reaching their full reproductive potential [2]. Sow lifetime productivity is influenced by genetic factors and is determined by both annual reproductive performance and productive life [3,4]. Improving PSL not only maximizes the reproductive capacity of sows but also reduces the cost per weaned piglet by spreading sow-related costs over more offspring [5,6]. Our previous studies showed that a large proportion of sow culling (52.89–62.75%) occurs in low parities (from replacement gilts to parity 2), which severely reduces lifetime productivity [7,8]. Therefore, optimizing gilt development is essential to decrease the culling rate of young sows and improve their lifetime reproductive performance.

It is widely accepted that the core goal of gilt development is to achieve early puberty onset and appropriate first insemination [9]. Numerous studies have confirmed that developmental traits at puberty and first insemination, including age, body weight, and backfat thickness, have substantial impacts on reproductive performance, first-parity productivity, and overall lifetime productivity of sows [10,11]. Given inherent genetic discrepancies among breeding lines, trait target values remain inconsistent. Although multiple breeding companies and research organizations have formulated line-specific reference targets for the above indicators, considerable disparities still exist between these proposed criteria. Such inconsistencies also lead to obvious differences in gilt rearing costs. Accordingly, this study was conducted to investigate the effects of key traits at puberty and first insemination on the long-term reproductive performance of DanBred sows, aiming to provide a scientific basis for optimizing gilt development programs and enhancing sow lifetime productivity.

## 2. Materials and Methods

### 2.1. Experimental Animals and Management

The experiment was conducted from October 2020 to June 2023 on a commercial swine farm in southern China. A total of 3025 replacement gilts (Landrace × Large White) and Landrace × (Landrace × Large White) at approximately 165 days of age were selected. The gilts were housed in group pens, with 15–20 animals per pen, providing a floor space of 1.5–2.0 m^2^ per pig. Estrus stimulation was initiated at 170 days of age via direct exposure to mature boars for 15–20 min daily. Estrus detection was performed daily using the back-pressure test combined with observation of vulval reddening and swelling. Gilts exhibiting a distinct standing reflex were defined as in estrus and recorded accordingly. In accordance with the DanBred(Vejle, Denmark) gilt management guidelines and standard farm protocol, gilts were typically inseminated at their second observed estrus. The decision to breed on the second estrus was made by farm management based on age, body weight, and backfat assessment. Artificial insemination was conducted 12 h after the onset of estrus, twice daily (morning and evening). Following insemination, sows were transferred to the gestation barn. Non-pregnant sows and those that returned to estrus were identified between 18 and 24 days post-insemination. Pregnancy diagnosis was carried out via ultrasound 30 days post-insemination, and to ensure accuracy, any sows diagnosed with pregnancy failure were re-examined one week later. Sows that returned to estrus or experienced early pregnancy failure were removed from the gestation unit, while pregnant sows were transferred to the farrowing house on day 109 of gestation.

Rearing feeding strategies were referenced to DanBred’s gilt management manual (https://danbred-manual.com/en/sow-gilt-manual/gilt-unit/ (accessed on 20 August 2020)). Gilts were fed 2.5–3.0 kg/day with ad libitum access to water. Flush feeding (approximately 3.5 kg/day) was applied for 10–14 days before insemination. A phase-feeding program was adopted during gestation: 1.8 kg/day on days 0–3, 2.2 kg/day on days 4–30, 2.4 kg/day on days 31–90, and 2.8 kg/day on days 91–110. Sows were fed ad libitum throughout lactation. The gestation diet contained 2050 kcal/kg DM net energy, 13% crude protein, and 0.60% standardized ileal digestible (SID) lysine. The lactation diet contained 2500 kcal/kg DM net energy, 18% crude protein, and 1.00% SID lysine.

### 2.2. Data Collection

All replacement gilts received standardized estrus stimulation twice daily in the morning and evening. Boar exposure combined with vulva observation and back-pressure reflex detection was applied for estrus identification. The age at the first estrus stimulation was accurately recorded. The age at puberty was defined as the day when the first standing estrus behavior was first observed. On the day of puberty onset, individual body weight was measured using an electronic scale under fasting conditions, and backfat thickness at the last rib was detected by a portable B-ultrasound instrument. The age at first insemination was recorded, and body weight and backfat thickness were re-measured with the same methods on the insemination day. All measurements were performed by unified operators with consistent equipment to guarantee data reliability. Data were collected for replacement gilts, including age at first estrus stimulation, age at puberty (first observed estrus), body weight and backfat thickness at puberty, as well as age, body weight and backfat thickness at first insemination. These parameters were grouped according to the criteria presented in Table 1. The estrus rate and breeding retention rate were calculated. For sows from parity 1 to 5, records related to insemination, farrowing, weaning, and reproductive performance were compiled. These included farrowing rate, total piglets born, piglets born alive, number of healthy piglets, and cumulative piglets weaned across five parities. Furthermore, sow culling and removal data were collected to determine sow retention rates. Culling decisions were made by farm management according to standard commercial criteria, including reproductive failure (e.g., return to estrus, abortion, non-pregnancy), lameness, low litter size, and poor body condition. Sows that failed to conceive after two consecutive inseminations or that exhibited severe health issues were removed from the herd. These criteria were applied consistently across all parities.

### 2.3. Statistical Analysis

Statistical analyses were performed using SAS software (version 9.4, SAS Institute, Cary, NC, USA). Prior to statistical modeling, multicollinearity diagnosis among all explanatory variables was conducted using Tolerance and Variance Inflation Factor (VIF). Variables with VIF < 10 and Tolerance > 0.1 were retained to exclude severe multicollinearity. Subsequently, multiple linear regression and mixed model analysis were performed. Continuous variables, including total piglets born, piglets born alive, number of healthy piglets, and piglets weaned across five parities, were analyzed using the PROC MIXED procedure. The fixed effects included age at puberty, body weight at puberty, backfat thickness at puberty, age at first insemination, body weight at first insemination, and backfat thickness at first insemination. Individual sow and housing unit were included as random effects. One-way analysis was adopted to screen preliminary differences among groups. Once significant differences were observed, Duncan’s multiple range test was applied for post hoc multiple comparisons. The categorical indicator of retention rate (0 = culled, 1 = retained) was further analyzed using the PROC GENMOD procedure with a binomial probability distribution and logit link function. Data are presented as means ± standard error of the mean (SEM), and significance was declared at *p* < 0.05.

## 3. Results

### 3.1. Descriptive Characteristics of Sows at Different Stages

As shown in Table 2, of the 3025 gilts included in the study, 2673 exhibited estrus and 2651 were successfully inseminated. The estrus rate and breeding retention rate were 88.36% and 87.63%, respectively. The farrowing rates for parities 1 to 5 were 90.91%, 93.93%, 91.93%, 92.38%, and 91.09%, while the corresponding retention rates were 85.10%, 73.82%, 59.37%, 47.42%, and 36.36%, respectively. The retention rate showed a consistent decline with increasing parity, decreasing by approximately 11–14 percentage points per parity from parity 1 to parity 5.

### 3.2. Pubertal and First-Insemination Parameters of Replacement Gilts

As shown in Table 3, the gilts had an average age at puberty (first observed estrus) of 209.87 days, with a corresponding average body weight of 140.57 kg and an average backfat thickness of 14.07 mm. Regarding the parameters at first insemination, the average age at first insemination was 235.00 days, accompanied by an average body weight of 153.09 kg and an average backfat thickness of 15.32 mm.

### 3.3. Associations Between Puberty and First Insemination Traits and Reproductive Outcomes in Sows

As shown in Table 4, multicollinearity diagnosis indicated no severe collinearity among predictors (all VIF < 10, Tolerance > 0.1), so all puberty and mating-related traits were retained for regression analysis.

Multiple linear regression was used to identify the most influential variables for reproductive performance and to verify linear responses. As shown in Table 5, multiple linear regression showed that age at puberty was significantly and negatively associated with total born, born alive, healthy piglets, and weaned piglets (*p* < 0.01), indicating that earlier puberty onset improved litter size outcomes across parities. Additionally, backfat thickness at first estrus and first insemination showed consistent positive effects on piglets born alive, healthy piglets, and weaned piglets (*p* < 0.05). Age at first insemination also positively influenced these traits (*p* < 0.05). In contrast, body weight at puberty and mating had limited or no significant effects on the measured reproductive indicators (*p* > 0.05).

### 3.4. Effect of Age at Puberty on the Reproductive Performance and Retention Rate of Sows over Five Parities

As shown in Table 6, gilts that reached puberty before 220 days of age produced significantly more total piglets born and total piglets live born over five parities compared with those that reached puberty after 220 days of age (*p* < 0.01). Furthermore, gilts that reached puberty between 190 and 219 days of age had significantly more healthy piglets and weaned piglets across five parities than those that reached puberty after 230 days of age (*p* < 0.01). The cumulative retention rate over five parities exhibited a declining trend with increasing age at puberty. Notably, gilts that reached puberty before 200 days of age had a significantly higher five-parity cumulative retention rate than those that reached puberty after 210 days of age (*p* < 0.01).

### 3.5. Effect of Body Weight at Puberty on the Reproductive Performance and Retention Rate of Sows over Five Parities

As presented in Table 7, body weight at puberty had no significant effect (*p* > 0.05) on the total number of piglets born, number of piglets born alive, number of healthy piglets, or five-parity cumulative retention rate.

### 3.6. Effect of Backfat Thickness at Puberty on the Reproductive Performance and Retention Rate of Sows over Five Parities

As shown in Table 8, the total number of piglets born, piglets born alive, and healthy piglets across five parities increased with increasing backfat thickness at puberty. Specifically, gilts with a backfat thickness of >15 mm at puberty had significantly more total piglets born and piglets born alive than the other two groups (*p* < 0.01). Furthermore, gilts with a backfat thickness of 14–15 mm had significantly more piglets born alive than those with <14 mm backfat thickness at puberty (*p* < 0.05). Regarding the total number of healthy piglets across five parities, the > 15 mm group had significantly more healthy piglets than the <14 mm group (*p* < 0.01). The total number of weaned piglets also increased significantly in gilts with > 15 mm backfat thickness at puberty (*p* < 0.01). The five-parity retention rate exhibited a positive trend with increasing backfat thickness at puberty, with the highest retention rate (40.18%) observed in gilts with >15 mm backfat thickness (*p* = 0.08).

### 3.7. Effect of Age at First Insemination on the Reproductive Performance and Retention Rate of Sows over Five Parities

As shown in Table 9, gilts inseminated at 210–219 days and 250–259 days of age produced significantly more total piglets across five parities than those inseminated before 210 days or at ≥260 days of age (*p* < 0.05), with no significant differences observed between the other groups. For the total number of piglets born alive over five parities, gilts inseminated at 250–259 days of age had significantly more piglets born alive than those inseminated before 210 days or at ≥260 days of age (*p* < 0.05), and no significant differences existed between the other groups. Regarding the total number of healthy piglets across five parities, gilts inseminated at 210–219 days and 240–259 days of age had significantly more healthy piglets than those inseminated before 210 days of age (*p* < 0.05), whereas no significant differences were detected between the other groups. Age at first insemination did not significantly affect the total number of piglets weaned over five parities (*p* = 0.10). However, the five-parity retention rate showed a significant decreasing trend with increasing age at first insemination (*p* < 0.01).

### 3.8. Effect of Body Weight at First Insemination on the Reproductive Performance and Retention Rate of Sows over Five Parities

As shown in Table 10, gilts with a body weight exceeding 150 kg at first insemination produced significantly more total piglets and healthy piglets across five parities than those weighing 130–139.9 kg (*p* < 0.01). Regarding the total number of piglets born alive, gilts with a body weight above 140 kg at first insemination had significantly more piglets born alive than the 130–139.9 kg group (*p* < 0.01). However, body weight at first insemination had no significant effect on the total number of piglets weaned or the five-parity retention rate (*p* > 0.05).

### 3.9. Effect of Backfat Thickness at First Insemination on the Reproductive Performance and Retention Rate of Sows over Five Parities

As shown in Table 11, although backfat thickness at first insemination had no significant effect on the five-parity retention rate (*p* > 0.05), gilts with a backfat thickness of >16 mm exhibited significantly greater total numbers of piglets born, piglets born alive, healthy piglets, and piglets weaned across five parities than those in the other two groups (*p* < 0.01).

## 4. Discussion

### 4.1. Evaluating the Effectiveness of Replacement Gilt Development

The main purpose of gilt development is to meet both the quantitative demand for herd replacement and the qualitative requirement for high reproductive performance [12]. Common indices for evaluating gilt development include estrus rate and first-parity reproductive performance, such as litter size, number of piglets weaned, and weaning-to-estrus interval [13]. Although litter size has low heritability, studies have shown a significant correlation between first-parity and subsequent-parity litter sizes. For example, sows with more than 15 piglets born alive in the first parity produce 0.5–1.8 more piglets per litter in later parities and have 2.8–5.4% higher farrowing rates across parities 1–3 than sows with smaller first-parity litters [4].

These traits are strongly influenced by genetics and gilt rearing practices, and major industry organizations have defined clear performance targets for first-parity sows. Faccin et al. (2022) recommends more than 11.8 piglets born in the first litter [14], PIC (2021) suggests ≥15.5 total piglets born and ≥14.5 piglets born alive [15], and the Topigs Norsvin Nutrition Team (2023) recommends more than 15 piglets born alive [16]. Although first-parity performance is closely related to long-term reproductive efficiency, it does not reflect culling rates, which are key factors affecting overall herd productivity.

The present results indicate that most sow culling occurs during early parities. Culling of gilts and sows in the first and second parities accounts for 62.76% of total sow removal, highlighting the importance of reducing early-parity culling rates [7]. For reference, PIC recommends retention rates of ≥95% for parity 1, ≥85% for parity 2, and ≥75% for parity 3. However, in commercial production systems, actual third-parity retention rates are usually between 20% and 60% [17]. In this study, the retention rates for the first three parities were 85.10%, 73.82%, and 59.37%, respectively, which are below the recommended targets. The lower retention rates of the first three parities in this study are mainly attributed to genetics, gilt rearing management and intensive commercial production. These results indicate the need to develop more accurate and science-based indicators to effectively evaluate and improve gilt development programs.

### 4.2. Effect of Pubertal Parameters on Lifetime Reproductive Performance

Puberty onset is a critical milestone for gilts to gain reproductive capacity [18]. Key indicators including age, body weight, and backfat thickness at puberty reflect gilt growth, maturity, and reproductive potential, and directly affect lifetime reproductive performance, including productive lifespan, total piglets born, conception rate, and culling rate [19]. Age at puberty is a reliable predictor of reproductive performance and longevity in sows [13], with moderate heritability ranging from 0.25 to 0.46 [17].

In the present study, multicollinearity was diagnosed and multiple linear regression was applied to distinguish the most critical variables. Age at puberty was identified as the most influential negative predictor, indicating that earlier puberty was consistently beneficial for long-term reproductive performance. Linear regression confirmed a significant linear reduction in litter size with delayed puberty, supporting the existence of an optimal early window for puberty onset.

The present study found that gilts reaching puberty before 220 days of age had higher total piglets born and piglets born alive across five parities. In addition, the retention rate decreased significantly as age at puberty increased. In a study of 1257 PIC gilts, Young et al. reported that gilts reaching puberty before 185 days had significantly higher total piglets born (27.3 vs. 24.8), piglets born alive (24.8 vs. 22.5), and piglets weaned (22.3 vs. 20.4) over three parities, as well as a significantly lower three-parity culling rate (38.3% vs. 47.5%), than those reaching puberty after 185 days [20]. Similarly, Patterson et al. analyzed 431 PIC gilts and found that gilts reaching puberty between 140 and 180 days had a significantly higher three-parity retention rate than those reaching puberty after 180 days (45.74% vs. 28.95%) [21]. Therefore, although age at puberty may vary with genetic background, early puberty is beneficial for improving lifetime reproductive output and multi-parity retention.

In this study, body weight at puberty had no significant effect on lifetime reproductive performance or five-parity retention. In contrast, gilts with higher backfat thickness at puberty showed better lifetime performance and higher retention rates. Body composition, especially backfat thickness, is associated with sexual maturity and estrus initiation. Increased body fat reserves raise leptin levels, activate the GnRH reproductive axis, and promote puberty onset [22]. Moreover, adequate body fat is essential for lactation performance and longevity once gilts enter the breeding herd [23]. These results align with the DanBred gilt development manual, which recommends targeted body condition rather than simple body weight for gilt management.

Therefore, although age at puberty may vary with genetic background, early puberty is beneficial for improving lifetime reproductive output and multi-parity retention. The multiple regression analysis in the present study further demonstrated that age at puberty was negatively associated with total born, born alive, healthy piglets, and weaned piglets, while backfat thickness at puberty showed positive associations with these outcomes. These findings support the conclusion that age and backfat thickness at puberty are not merely isolated predictors but interact to determine lifetime productivity. Rather than addressing these traits independently, an integrated approach monitoring both early puberty onset and adequate body fat deposition should be applied in gilt development programs to optimize long-term sow performance.

### 4.3. Effect of First-Insemination Parameters on Lifetime Reproductive Performance

First insemination is a critical management decision that affects both rearing costs and lifetime productivity in gilts. It is generally recommended that gilts be bred at their second or later estrus cycle if they have cycled before 200 d of age [24]. Our previous research indicated that breeding gilts at the third or later estrus cycle did not improve reproductive performance compared with breeding at the second estrus, but increased feeding costs [8]. In addition, Saito et al. analyzed data from 38,212 Japanese sows (2001–2006) and found that as age at first insemination increased from 240 to 257 days, the number of piglets born alive per sow per year decreased from 18.5 to 15.3, and lifetime parities declined from 4.9 to 4.1 [10].

The present study demonstrated that gilts inseminated between 210 and 260 days of age had better reproductive performance across five parities. However, the five-parity retention rate decreased significantly with increasing age at first insemination. Therefore, considering feed efficiency, lifetime productivity, and retention rates, early first insemination within an appropriate window is generally advantageous.

Body weight at first insemination also significantly affects lifetime reproductive performance. Although the present study found no effect of body weight at first insemination on five-parity retention or total piglets weaned, gilts weighing over 150 kg had larger litter sizes. However, controlling gilt body weight at breeding within an appropriate range is still crucial. Amaral Filha et al. found that sows bred at weights over 160 kg had significantly fewer lifetime total pigs born. Similarly, breeding gilts weighing below 135 kg or above 170 kg resulted in significantly fewer piglets born alive in the first three parities [25]. Body weight is also closely related to body composition.

The effect of backfat thickness at first insemination on lifetime performance remains inconsistent in current research. Multiple linear regression revealed that backfat thickness at first insemination was a major positive predictor of litter size across parities, with a linear increase in performance as backfat increased. An optimal threshold of >16 mm backfat was identified, beyond which reproductive performance was maximized. Age at first insemination also positively affected litter traits in the regression model, but retention rate declined linearly with increasing age, demonstrating a trade-off between litter size and herd longevity. Tummaruk et al. suggested that backfat thickness at puberty does not affect litter performance [26], while Amaral Filha et al. reported that sows with 16–17 mm backfat thickness had the highest total number of piglets born and born alive [27]. In this study, although backfat thickness at first insemination did not affect five-parity retention, gilts with >16 mm backfat thickness at first insemination had significantly more total piglets born, born alive, healthy piglets, and piglets weaned across five parities than the other groups.

The multiple regression analysis conducted in this study confirmed that backfat thickness at first insemination was positively and consistently associated with piglets born alive, healthy piglets, and weaned piglets, while age at first insemination showed a more complex pattern beneficial within an optimal window but detrimental to retention when excessively delayed. These results highlight the value of multivariate evaluation over univariate comparisons, as the former better captures the interdependent nature of developmental parameters and their combined effects on lifetime productivity.

## 5. Conclusions

In conclusion, pubertal and first-insemination parameters play pivotal roles in determining the lifetime reproductive performance and retention rates of sows. Multicollinearity diagnosis and multiple linear regression confirmed key predictors, linear responses, and optimal thresholds. Gilts that attain puberty at 190–199 days of age with a backfat thickness of >15 mm exhibit superior reproductive outcomes and higher five-parity retention rates. For first insemination, gilts mated at second estrus (210–229 days), with a body weight of ≥140 kg and a backfat thickness of >16 mm, show significantly improved lifetime reproductive performance across five parities. The optimal puberty and first-insemination parameters obtained from this study can provide a reference standard for the rearing of DanBred replacement gilts, fully exploit the reproductive potential of DanBred sows, and thereby improve the reproductive efficiency and economic benefits of large-scale pig farms. Overall, the results suggest that implementing the DanBred gilt management guidelines yields satisfactory reproductive outcomes in this commercial herd, though further optimization of age and body condition targets within the recommended ranges can enhance lifetime productivity and sow retention.

## Figures and Tables

**Table 1:** Grouping of replacement gilt development parameters.

No.	Breeding Parameters	Group
1	Age at puberty, d	<190, 190–199, 200–209, 210–219, 220–229, 230–239, ≥240
2	Body weight at puberty, kg	<115, 115–124.9, 125–134.9, 135–144.9, 145–154.9, 155–164.9, ≥165
3	Backfat thickness at puberty, mm	<14, 14–15, ≥16
4	Age at first insemination, d	<210, 210–219, 220–229, 230–239, 240–249, 250–259, ≥260
5	Body weight at first insemination, kg	<130, 130–139.9, 140–149.9, 150–159.9, 160–169.9, 170–179.9, ≥180
6	Backfat thickness at first insemination, mm	<15, 15–16, >16

**Table 2:** Basic information of sows.

Item		Size	Estrus Rate, %	Breeding Retention Rate, %	Farrowing Rate, %	Retention Rate, %
Breeding stage	Herd	3025	88.36	87.63		
	Puberty	2673				
Parity 1	Breeding	2651			90.91	85.10
	Farrowing	2410				
	Weaning	2378				
Parity 2	Breeding	2256			93.93	73.82
	Farrowing	2119				
	Weaning	2110				
Parity 3	Breeding	1957			91.93	59.37
	Farrowing	1799				
	Weaning	1777				
Parity 4	Breeding	1574			92.38	47.42
	Farrowing	1454				
	Weaning	1424				
Parity 5	Breeding	1257			91.09	36.36
	Farrowing	1145				
	Weaning	1127				
Parity 6	Breeding	964				

**Table 3:** Basic information of rearing parameters in replacement gilts.

Breeding Parameters	Parameter Information	Parameter Range
Age at puberty, d	210 ± 1	163–317
Body weight at puberty, kg	141.6 ± 0.3	87–195
Backfat thickness at puberty, mm	14.1 ± 0.1	8–23
Age at first insemination, d	235 ± 1	188–318
Body weight at first insemination, kg	153.1 ± 0.3	120–207
Backfat thickness at first insemination, mm	15.3 ± 0.1	8–27

Data are presented as means ± standard error of the mean (SEM).

**Table 4:** Multicollinearity diagnosis of puberty and first insemination-related variables.

Variable	VIF	Tolerance
Age at puberty	2.15	0.47
Body weight at puberty	1.72	0.58
Backfat thickness at puberty	1.88	0.53
Age at first insemination	2.28	0.44
Body weight at first insemination	1.93	0.52
Backfat thickness at first insemination	1.60	0.62

VIF, variance inflation factor. Variables with VIF < 10 and Tolerance > 0.1 were considered free of severe multicollinearity. Tolerance = 1 − R^2^_i_, where R^2^_i_ is the coefficient of determination of the regression of variable i on the remaining predictors.

**Table 5:** Multiple linear regression analysis of puberty and first insemination traits on reproductive performance across five parities in sows.

Response	Variable	β	SE	t	*p* Value
Average total number of piglets born	Age at puberty	−0.12	0.02	−5.23	<0.01
	Body weight at puberty	0.06	0.03	2.11	0.04
	Backfat thickness at puberty	0.40	0.19	2.08	0.04
	Age at first insemination	0.04	0.03	1.44	0.15
	Body weight at first insemination	0.05	0.03	1.52	0.13
	Backfat thickness at first insemination	0.26	0.18	1.44	0.15
Average total number of piglets born alive	Age at puberty	−0.14	0.02	−6.28	<0.01
	Body weight at puberty	0.04	0.03	1.49	0.14
	Backfat thickness at puberty	0.39	0.19	2.01	0.04
	Age at first insemination	0.06	0.03	2.43	0.02
	Body weight at first insemination	0.06	0.03	1.71	0.09
	Backfat thickness at first insemination	0.45	0.18	2.51	0.01
Average total number of healthy piglets	Age at puberty	−0.10	0.02	−5.10	<0.01
	Body weight at puberty	0.02	0.02	0.96	0.33
	Backfat thickness at puberty	0.24	0.16	1.52	0.13
	Age at first insemination	0.07	0.02	3.11	<0.00
	Body weight at first insemination	0.00	0.03	−0.14	0.89
	Backfat thickness at first insemination	0.38	0.15	2.54	0.01
Average total number of piglets weaned	Age at first estrus	−0.11	0.02	−6.86	0.00
	Body weight at first estrus	0.03	0.02	1.55	0.12
	Backfat thickness at first estrus	0.11	0.14	0.78	0.43
	Age at first insemination	0.04	0.02	2.03	0.04
	Body weight at first insemination	−0.04	0.02	−1.57	0.12
	Backfat thickness at first insemination	0.43	0.13	3.37	<0.01

β, standardized regression coefficient; SE, standard error of the regression coefficient; t, *t*-test statistic; *p* < 0.05 or *p* < 0.01 indicate significant difference.

**Table 6:** Effects of age at puberty on reproductive performance and retention rate of sows over five parities.

Age	<190	190–199	200–209	210–219	220–229	230–239	≥240	SEM	*p* Value
Size	96	232	249	157	106	42	82		
Average total number of piglets born	78.51 ^a^	78.80 ^a^	78.08 ^a^	77.90 ^a^	74.98 ^b^	73.93 ^b^	72.82 ^b^	0.31	<0.01
Average total number of piglets born alive	74.63 ^a^	75.53 ^a^	75.48 ^a^	75.31 ^a^	71.22 ^b^	69.17 ^b^	68.46 ^b^	0.28	<0.01
Average total number of healthy piglets	65.83 ^ab^	66.30 ^a^	67.09 ^a^	67.20 ^a^	64.63 ^ab^	61.79 ^b^	61.87 ^b^	0.26	<0.01
Average total number of piglets weaned	62.56 ^ab^	63.58 ^a^	64.67 ^a^	64.32 ^a^	62.57 ^ab^	59.64 ^bc^	55.69 ^c^	0.25	<0.01
Retention rate, %	41.38 ^a^	43.53 ^a^	38.73 ^ab^	30.60 ^c^	36.81 ^b^	36.52 ^b^	29.71 ^c^	0.12	<0.01

Values within the same column with the same or no superscript letters indicate no significant difference (*p* > 0.05), while different superscript letters indicate significant difference at *p* < 0.05 or *p* < 0.01. SEM, standard error of the mean.

**Table 7:** Effect of body weight at puberty on reproductive performance and retention rate of sows over five parities.

Body Weight	<115	115–124.9	125–134.9	135–144.9	145–154.9	155–164.9	≥165	SEM	*p* Value
Size	41	123	195	209	198	126	72		
Average total number of piglets born	74.93	75.10	76.90	77.71	78.81	77.72	77.39	0.27	0.33
Average total number of piglets born alive	71.85	72.37	73.92	74.30	75.52	74.20	73.33	0.26	0.46
Average total number of healthy piglets	64.98	65.18	66.25	65.97	66.34	65.61	65.08	0.25	0.95
Average total number of piglets weaned	62.71	63.59	63.07	62.68	63.01	63.31	61.22	0.24	0.31
Retention rate, %	37.27	34.55	34.64	37.12	38.37	39.74	36.36	0.10	0.70

SEM, standard error of the mean.

**Table 8:** Effect of backfat thickness at puberty on reproductive performance and retention rate of sows over five parities.

Backfat Thickness	<14	14–15	>15	SEM	*p* Value
Size	427	316	221		
Average total number of piglets born	75.53 ^c^	77.74 ^b^	80.09 ^a^	0.31	<0.01
Average total number of piglets born alive	72.14 ^c^	74.41 ^b^	77.17 ^a^	0.29	<0.01
Average total number of healthy piglets	64.71 ^b^	66.24 ^ab^	67.48 ^a^	0.27	<0.01
Average total number of piglets weaned	62.42 ^b^	62.63 ^b^	64.29 ^a^	0.26	<0.01
Retention rate, %	34.83	37.40	40.18	0.10	0.08

Values within the same column with the same or no superscript letters indicate no significant difference (*p* > 0.05), while different superscript letters indicate significant difference at *p* < 0.05 or *p* < 0.01. SEM, standard error of the mean.

**Table 9:** Effect of age at first insemination on reproductive performance and retention rate of sows over five parities.

Age	<210	210–219	220–229	230–239	240–249	250–259	≥260	SEM	*p* Value
Size	35	183	241	155	139	97	114		
Average total number of piglets born	75.60 ^b^	78.91 ^a^	77.33 ^ab^	76.79 ^ab^	76.11 ^ab^	79.77 ^a^	75.21 ^b^	0.30	0.02
Average total number of piglets born alive	71.49 ^b^	75.16 ^ab^	73.82 ^ab^	73.47 ^ab^	73.75 ^ab^	76.91 ^a^	72.15 ^b^	0.29	0.03
Average total number of healthy piglets	62.80 ^b^	66.20 ^a^	65.78 ^ab^	65.42 ^ab^	66.12 ^a^	67.55 ^a^	65.13 ^ab^	0.27	0.02
Average total number of piglets weaned	62.17	62.36	63.95	63.45	63.14	62.80	60.97	0.26	0.10
Retention rate, %	47.30 ^a^	41.97 ^ab^	40.78 ^ab^	38.75 ^bc^	36.39 ^bc^	32.22 ^cd^	25.73 ^d^	0.11	<0.01

Values within the same column with the same or no superscript letters indicate no significant difference (*p* > 0.05), while different superscript letters indicate significant difference at *p* < 0.05 or *p* < 0.01. SEM, standard error of the mean.

**Table 10:** Effect of body weight at first insemination on reproductive performance and retention rate of sows over five parities.

Body Weight	<130	130–139.9	140–149.9	150–159.9	160–169.9	170–179.9	≥180	SEM	*p* Value
Size	29	128	259	248	167	92	41		
Average total number of piglets born	76.45 ^ab^	73.80 ^b^	76.22 ^ab^	78.22 ^a^	77.87 ^a^	80.29 ^a^	80.98 ^a^	0.32	<0.01
Average total number of piglets born alive	71.55 ^ab^	69.79 ^b^	73.04 ^a^	75.15 ^a^	75.04 ^a^	77.12 ^a^	77.61 ^a^	0.30	<0.01
Average total number of healthy piglets	66.14 ^ab^	62.64 ^b^	65.53 ^ab^	66.72 ^a^	66.52 ^a^	67.35 ^a^	66.22 ^a^	0.28	<0.01
Average total number of piglets weaned	60.28	62.35	63.09	63.42	63.39	61.61	63.44	0.27	0.09
Retention rate, %	37.66	36.16	40.03	34.21	35.53	37.86	39.42	0.12	0.44

Values within the same column with the same or no superscript letters indicate no significant difference (*p* > 0.05), while different superscript letters indicate significant difference at *p* < 0.05 or *p* < 0.01. SEM, standard error of the mean.

**Table 11:** Effect of backfat thickness at first insemination on reproductive performance and retention rate of sows over five parities.

Backfat Thickness	<15	15–16	>16	SEM	*p* Value
Size	370	343	251		
Average total number of piglets born	75.91 ^b^	76.46 ^b^	80.48 ^a^	0.31	<0.01
Average total number of piglets born alive	72.10 ^b^	73.29 ^b^	77.90 ^a^	0.30	<0.01
Average total number of healthy piglets	64.19 ^b^	65.78 ^b^	68.37 ^a^	0.30	<0.01
Average total number of piglets weaned	61.90 ^b^	62.03 ^b^	65.62 ^a^	0.28	<0.01
Retention rate, %	35.51	37.69	37.52	0.15	0.61

Values within the same column with the same or no superscript letters indicate no significant difference (*p* > 0.05), while different superscript letters indicate significant difference at *p* < 0.05 or *p* < 0.01. SEM, standard error of the mean.

## Data Availability

The raw data supporting the conclusions of this article will be made available by the authors upon request.

## References

[1] Sanz-Fernández S., Rodríguez-Hernández P., Díaz-Gaona C., Tusell L., Quintanilla R., Rodríguez-Estévez V. (2024). Evolution of Sow Productivity and Evaluation Parameters: Spanish Farms as a Benchmark. Vet. Sci..

[2] Tani S., Piñeiro C., Koketsu Y. (2018). Culling in Served Females and Farrowed Sows at Consecutive Parities in Spanish Pig Herds. Porc. Health Manag..

[3] Koketsu Y., Iida R. (2020). Farm Data Analysis for Lifetime Performance Components of Sows and Their Predictors in Breeding Herds. Porc. Health Manag..

[4] Iida R., Piñeiro C., Koketsu Y. (2015). High Lifetime and Reproductive Performance of Sows on Southern European Union Commercial Farms Can Be Predicted by High Numbers of Pigs Born Alive in Parity One. J. Anim. Sci..

[5] Tani S., Piñeiro C., Koketsu Y. (2018). High-Performing Farms Exploit Reproductive Potential of High and Low Prolific Sows Better than Low-Performing Farms. Porc. Health Manag..

[6] Ordaz G., López M., Pérez R.E., Mariscal G., Ortiz R. (2024). Factors associated with the productive longevity of sows in commercial breeding herds. Arch. Anim. Breed..

[7] Wang C., Wu Y., Shu D., Wei H., Zhou Y., Peng J. (2019). An Analysis of Culling Patterns during the Breeding Cycle and Lifetime Production from the Aspect of Culling Reasons for Gilts and Sows in Southwest China. Anim. Open Access J..

[8] Tan J., Wang M., Sun H., Wang C., Wei H., Jiang S., Zhou Y., Peng J. (2023). Optimizing Feeding Regimen of Replacement Gilts to Improve Their Reproductive Performance and Retention Rate of Their First 2 Parities. Anim. Nutr..

[9] Kummer R., Bernardi M.L., Wentz I., Bortolozzo F.P. (2006). Reproductive Performance of High Growth Rate Gilts Inseminated at an Early Age. Anim. Reprod. Sci..

[10] Saito H., Sasaki Y., Koketsu Y. (2011). Associations between Age of Gilts at First Mating and Lifetime Performance or Culling Risk in Commercial Herds. J. Vet. Med. Sci..

[11] Sasaki Y., Tokunaga T., Uemura R., Sueyoshi M. (2014). An Assessment of Reproductive and Lifetime Performances of Kagoshima Berkshire Gilts and Sows. Anim. Sci. J. Nihon Chikusan Gakkaiho.

[12] Zhuo Y., Hua L., Che L., Fang Z., Lin Y., Xu S., Wang J., Li J., Feng B., Wu D. (2022). Dietary Fiber Supplementation in Replacement Gilts Improves the Reproductive Performance From the Second to Fifth Parities. Front. Vet. Sci..

[13] Rohrer G., Cross A., Lents C., Miles J., Nonneman D., Rempel L. (2017). 026 Genetic Improvement of Sow Lifetime Productivity. J. Anim. Sci..

[14] Faccin J.E.G., Tokach M.D., Goodband R.D., DeRouchey J.M., Woodworth J.C., Gebhardt J.T. (2022). Gilt Development to Improve Offspring Performance and Survivability. J. Anim. Sci..

[15] PIC (2021). Gilt & Sow Management Manual. https://www.pic.com/resources/gilt-sow-management-guidelines-english.

[16] Topigs Norsvin Nutrition Team TN70 FEED MANUAL. https://norsvin.no/wp-content/uploads/2023/08/Manual-TN70-HR-2023.pdf.

[17] Li Q., Yuan X., Chen Z., Zhang A., Zhang Z., Zhang H., Li J. (2018). Heritability Estimates and Effect on Lifetime Reproductive Performance of Age at Puberty in Sows. Anim. Reprod. Sci..

[18] Roongsitthichai A., Cheuchuchart P., Chatwijitkul S., Chantarothai O., Tummaruk P. (2013). Influence of Age at First Estrus, body Weight, and Average Daily Gain of Replacement Gilts on Their Subsequent Reproductive Performance as Sows. Livest. Sci..

[19] Patterson J., Foxcroft G. (2019). Gilt Management for Fertility and Longevity. Anim. Open Access J..

[20] Young B., Dewey C.E., Friendship R.M. (2010). Management Factors Associated with Farrowing Rate in Commercial Sow Herds in Ontario. Can. Vet. J. Rev. Vet. Can..

[21] Patterson J.L., Beltranena E., Foxcroft G.R. (2010). The Effect of Gilt Age at First Estrus and Breeding on Third Estrus on Sow Body Weight Changes and Long-Term Reproductive Performance. J. Anim. Sci..

[22] Barb C.R., Hausman G.J., Lents C.A. (2008). Energy Metabolism and Leptin: Effects on Neuroendocrine Regulation of Reproduction in the Gilt and Sow. Reprod. Domest. Anim. Zuchthyg..

[23] Strathe A.V., Bruun T.S., Hansen C.F. (2017). Sows with High Milk Production Had Both a High Feed Intake and High Body Mobilization. Anim. Int. J. Anim. Biosci..

[24] Kraeling R.R., Webel S.K. (2015). Current Strategies for Reproductive Management of Gilts and Sows in North America. J. Anim. Sci. Biotechnol..

[25] Amaral Filha W.S., Bernardi M.L., Wentz I., Bortolozzo F.P. (2010). Reproductive Performance of Gilts According to Growth Rate and Backfat Thickness at Mating. Anim. Reprod. Sci..

[26] Tummaruk P., Tantasuparuk W., Techakumphu M., Kunavongkrit A. (2009). The Association between Growth Rate, Body Weight, Backfat Thickness and Age at First Observed Oestrus in Crossbred Landrace x Yorkshire Gilts. Anim. Reprod. Sci..

[27] Williams N., Patterson J., Foxcroft G. (2005). Non-Negotiables of Gilt Development. Adv. Pork Prod..

